# Legal design for establishing a novel parastatal public health institute – Japan Institute for Health Security: A case study

**DOI:** 10.1371/journal.pgph.0007088

**Published:** 2026-08-26

**Authors:** Miyako Otsuka, Mio Kato, Takaji Wakita, Tomoya Saito

**Affiliations:** 1 Center for Emergency Preparedness and Response, National Institute of Infectious Diseases, Japan Institute for Health Security, Tokyo, Japan; 2 Japan Institute for Health Security, Tokyo, Japan; University of North Carolina at Chapel Hill, UNITED STATES OF AMERICA

## Abstract

The COVID-19 pandemic exposed critical gaps in Japan’s infectious disease governance, including fragmented structures and limited coordination between research and policy. In response, Japan established the Japan Institute for Health Security (JIHS) in April 2025 by merging the National Institute of Infectious Diseases and the National Center for Global Health and Medicine, yet no comprehensive assessment exists of the legal frameworks supporting this transformation. This descriptive qualitative study combined document review with semi-structured key informant interviews to examine JIHS governance structures, objectives, and legal reforms. We reviewed five pieces of legislation, two grey literature documents, and eight government publications, and interviewed eight purposively selected informants—one national legislator, four senior Ministry of Health, Labour and Welfare officials, and three JIHS executive officers—in Japanese between August and September 2025, analyzing transcripts with Braun and Clarke’s thematic analysis framework. The JIHS Act and associated amendments established JIHS as a Special Public Corporation, creating mechanisms for vertical and horizontal coordination and embedding preparedness and research capacity as core elements of national health security. Consistent with Japanese enabling legislation, the Act sets a broad framework whose operational adequacy is calibrated through subordinate regulations and administrative practice. Document analysis and interviews revealed a tripartite governance model integrating political leadership, administrative coordination, and scientific expertise, and informants emphasized that inter-agency trust and cultural integration between the merged institutions are essential for effective implementation. JIHS thus internalizes scientific advice within the national public health framework while preserving institutional autonomy, offering context-dependent lessons that underscore the importance of tailoring reforms to national administrative traditions. Future evaluation will focus on the real-world operationalization of this enacted framework, assessing whether its specific design choices translate into improved pandemic preparedness and response.

## Introduction

The Coronavirus Disease 2019 (COVID-19) pandemic, which emerged in late 2019, subjected nations across the globe to unprecedented challenges. Healthcare systems in many countries were overwhelmed by the rapid surge in patient numbers, resulting in significant increases in mortality. This global crisis underscored the urgent necessity of establishing comprehensive societal mechanisms to ensure effective responses to public health emergencies. In Japan, it has been noted that during the initial phase of the COVID-19 pandemic, the collection and analysis of patient data, clinical information, and epidemiological specimens were insufficient, hindering the government’s ability to implement timely and evidence-based countermeasures [1–3]. Moreover, the absence of an institutional mechanism linking clinical experts with policymakers resulted in fragmented responses across medical institutions and universities, limiting the rapid sharing of patient data and scientific insights [4]. This lack of a centralized command structure and the uncoordinated dispersal of responsibilities have been identified as major shortcomings in Japan’s crisis response framework [4]. Drawing on the lessons learned in Japan following the COVID-19 pandemic, this paper explores the establishment of the Japan Institute for Health Security (JIHS) to describe strategies for strengthening public health governance and emergency preparedness through legal and institutional reform.

## Background

Policy analysis by a research institute pointed out that Japan lagged behind other nations in the development, clinical trials, and production of vaccines and therapeutic agents during the pandemic [1]. The same analysis emphasized the necessity of strengthening systems that facilitate the collection and application of clinical data from designated infectious disease hospitals nationwide to inform more effective public health measures [1,4]. Recognising these lessons, Japan identified the need to strengthen its preparedness, response, and coordination capacities for future health crises. In light of this, the Government of Japan passed acts to establish the JIHS through the integration of two existing national institutions: the National Institute of Infectious Diseases (NIID) and the National Center for Global Health and Medicine (NCGM). It is worth noting a structural feature of the Japanese legislative system that bears directly on this inquiry. Statutes enacted by the Diet establish the basic framework, authorities, and principles, while the operational details are delegated to subordinate legislation (cabinet and ministerial ordinances, notifications) and to internal regulations and operational guidance.

The following section provides an overview of the two merging institutes.

### National Institute of Infectious Diseases (NIID).

The origins of NIID date back more than 130 years, when it was first founded as a private institute attached to the Hygiene Society of Japan in 1892. While it has undergone several name and jurisdiction changes over its history, NIID has consistently played a central role in infectious disease research in Japan. Following World War II, in 1947, the institute was formally attached to the Ministry of Health and Welfare and tasked with conducting (i) fundamental and applied research on infectious diseases and (ii) national testing for lot release and development of antibiotics and vaccines. NIID has undergone several subsequent reforms to its scope, including in 1997 the incorporation of leprosy research as well as national infectious disease surveillance activities into the institute’s remit [5].

During the COVID-19 pandemic, NIID played a central technical role in Japan’s response. The 2023 institutional evaluation report noted major contributions in: research and development, including establishing a Therapeutics and Vaccine Development Center; national surveillance and data dissemination; technical support to local public health institutes; international collaboration; and risk communication to the public. However, the institution faced constraints in budget, staffing, and structure. The 2023 report highlighted the need to improve risk communication, expand basic and clinical research capacity to generate credible evidence, and strengthen outbreak information-gathering and prediction capabilities – including use of mathematical models and Artificial Intelligence (AI) [6].

### National Center for Global Health and Medicine (NCGM).

NCGM was initially established as a military hospital in 1868. In 1945, the Ministry of Health and Welfare took responsibility for the NCGM’s activities. Over the latter half of the 20th century, the institute’s clinical functions for infectious diseases were expanded beyond the Center Hospital, into clinical research, international cooperation, nursing, and disease control and prevention [7].

During the COVID-19 pandemic, NCGM played critical clinical roles in Japan, including receiving evacuees from Wuhan, China and creating guidelines for their treatment and care.

Table 1 displays the roles and functions of NIID and NCGM immediately prior to the merger.

**Table 1:** Roles and functions of NIID and NCGM.

Institution	Main Functional Areas	Description
**National Institute of Infectious Diseases (NIID)** [5]	**1. Research Activities**	Conducts both basic and applied scientific research on infectious diseases, including the development of rapid diagnostic methods and vaccines.
	**2. Reference Services**	Maintains laboratory diagnostic systems and assays; stores and shares pathogen samples; standardizes reagents; prepares reference materials for disease diagnosis and surveillance; provides training and information sharing.
	**3. Surveillance Activities**	Operates Japan’s national infectious disease surveillance program, collecting, analyzing, and disseminating information from prefectural public health institutes and sentinel clinics under the Infectious Diseases Control Act. Conducts epidemiological investigations and information sharing during outbreaks.
	**4. Training**	Implements the Field Epidemiology Training Program (FETP) to build national capacity for epidemiological investigations and provides specialized technical training for international trainees on diseases such as AIDS, poliomyelitis, and leprosy.
	**5. National Certification and Testing**	Ensures the quality, efficacy, and safety of biological products and antibiotics through national lot release, performs government-mandated and client-requested tests, and establishes standards and reference materials for testing.
	**6. International Activities**	Promotes global collaboration with WHO and foreign research institutions through information dissemination, joint research, and technology transfer related to emerging and re-emerging infectious diseases.
	**7. Public Communication**	Engages with the public through outreach activities such as NIID Open Day to enhance understanding of the institute’s mission and research goals.
**National Center for Global Health and Medicine (NCGM)** [7]	**1. Center Hospital**	Serves as a national center for advanced and specialized medical care, addressing infectious diseases, metabolic disorders such as diabetes, and other chronic and intractable conditions in Japan’s aging society.
	**2. Center for Clinical Sciences**	Comprises six departments, Data Science, Clinical Research, International Trials, Clinical Research Strategic Planning, Regulatory Science, and Epidemiology and Prevention, advancing clinical and translational research.
	**3. Kohnodai Hospital**	A general hospital serving the local community and providing advanced medical care, research, and training related to hepatitis, immune diseases, and other conditions, both domestically and internationally.
	**4. Research Institute**	Functions as the core of NCGM’s research division and serves as Japan’s central base for international healthcare contributions.
	**5. Bureau of International Health Cooperation**	Works with MHLW, the Ministry of Foreign Affairs, JICA, WHO, and other organisations to improve global healthcare and public health, contributing to a healthier global society.
	**6. National College of Nursing**	Trains nursing professionals for the National Center for Advanced and Specialized Medical Care, cultivating nurses and midwives with clinical expertise aligned with national health policy.

### JIHS establishment.

**Fig 1:**
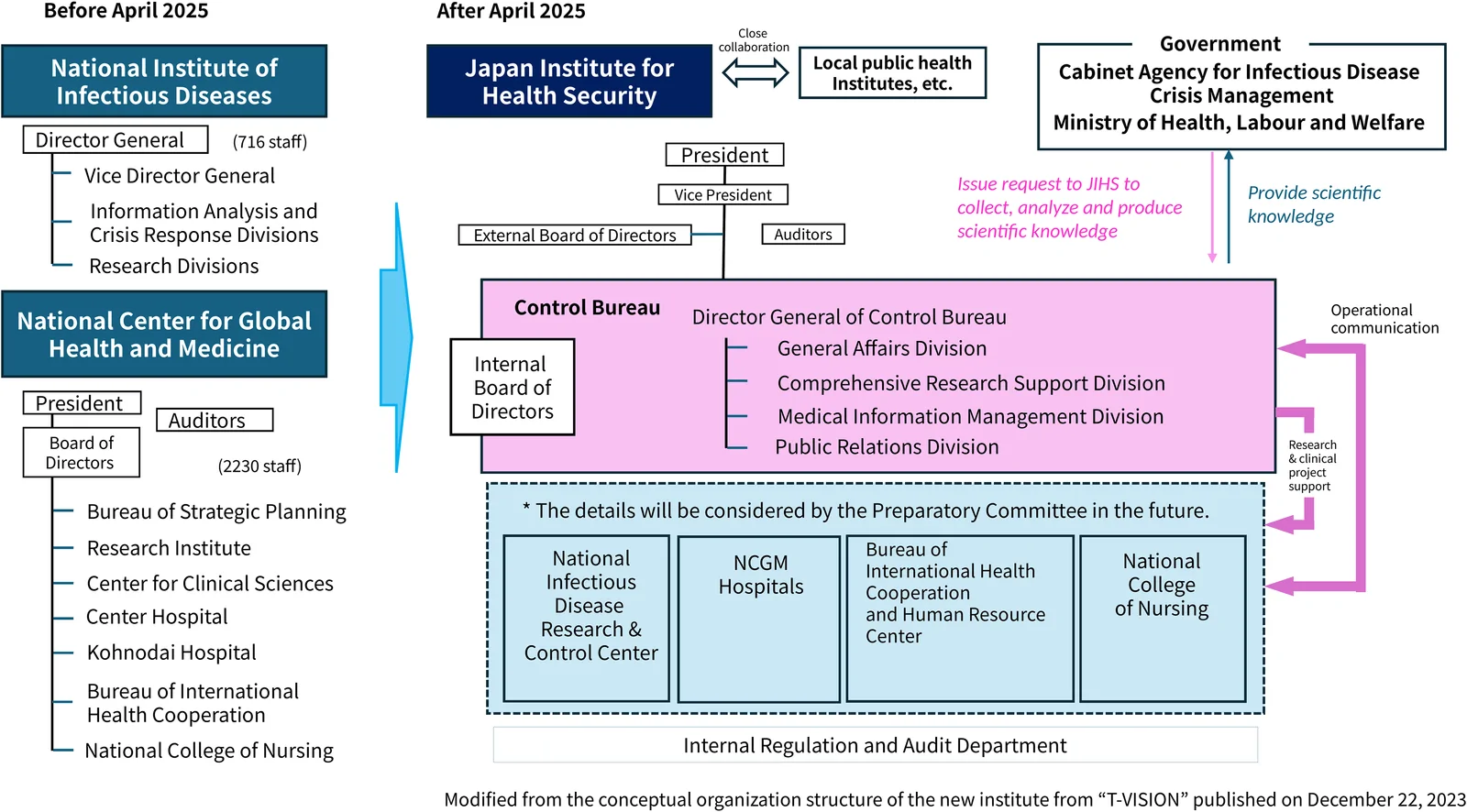
A comprehensive conceptual framework for JIHS through the merger of NIID and NCGM.

Upon its establishment through the integration of two preexisting institutions, JIHS was mandated to serve as a central body addressing the challenges previously identified, while capitalizing on the complementary functions of its predecessors. At the time of the legislation’s adoption, the institutional design of JIHS had not yet been concretely delineated; its specific functions and operational responsibilities were subsequently elaborated through subordinate legislation and operational guidance following the law’s passage. The description below presents the overarching conceptual framework of JIHS as it was initially conceived during this formative stage (Fig 1).

The need for legal frameworks when establishing public health institutions has been emphasized in international organisations and other countries around the world [8,9], and although the regions and cultures differ, the importance of verifying such legal frameworks prior to their use in emergencies is clear. Legal preparedness is considered a foundational element of effective health crisis management. Legal preparedness, as defined by the Global Health Security Agenda Legal Preparedness Action Package (GHSA LP AP), refers to the capacity to develop, assess, and implement legal frameworks that enable effective public health emergency preparedness and response [10,11]. This definition was formally adopted following extensive international consultation and is now widely used by governments, academics, and civil society organisations globally. The GHSA LP AP identifies five core elements of legal preparedness: 1. legal analysis—comprising legal mapping, legal assessment, and legal surveillance; 2. legal frameworks; 3. capacity; 4. coordination; and 5. implementation tools. This understanding is further reflected in the WHO Joint External Evaluation (JEE) Tool 3.0, which incorporates legal preparedness under the “Legal Instruments” technical area (Indicator P1), including the use of legal assessments and simulation exercises to test the adequacy of legal frameworks on a regular basis [12]. Grounding the analysis of the JIHS Act within these established frameworks enables a structured assessment of its strengths and gaps and offers lessons relevant both for Japan and for other countries seeking to strengthen their legal preparedness capacity.

The establishment of JIHS was preceded by government review processes that identified the institutional and legal shortcomings exposed by Japan’s COVID-19 response, and the JIHS Act was developed on the basis of these findings. The design challenge we identify does not, therefore, concern the adequacy of the legislative process itself, but rather whether the reformed framework proves operational and effective in practice—a question that has not yet been empirically examined. To date, no comprehensive assessment exists of whether all necessary legal authorities have been appropriately elaborated through the subordinate legislation and operational arrangements that give effect to the statutory framework, underscoring the need for the present study.

Against this background, the present study seeks to improve understanding of the new governance arrangements for, and objectives of, Japan’s health security system. This includes a. Understanding the functions of JIHS, b. Mapping these functions to the stated goals of reforming Japan’s health security system, and c. Identifying the law reforms conducted, including new laws and amendments to existing laws, to establish JIHS. Through this analysis, we aim to contribute to the broader discourse on legal and institutional readiness for public health emergencies both in Japan and globally.

## Methods

### Ethics statement

Ethical approval for this study was granted by the Japan Institute for Health Security (JIHS) Institutional Review Board (Approval No. JIHS-S-005089-00). Written informed consent was obtained from all participants prior to their interviews. All data were anonymized and securely stored in accordance with the Declaration of Helsinki.

This is a descriptive qualitative study, involving a literature review and key informant interviews to consolidate information from both publicly available sources and practitioner knowledge.

### Key document review

We reviewed publicly available documents from search engines and national Diet (Japan’s national legislature) library database, including legislation, grey literature, and government documents, to identify the motivations for reform, desired health security system functions, and the governance structures and law reforms related to the merger of NIID and NCGM and the establishment of JIHS. We then reviewed the identified legislation to understand changes to governance, function, and objectives of Japan’s health security system, including determining whether relevant authorities and powers are appropriately clear and well-defined.

### Key informant interview

This qualitative study used semi-structured key informant interviews to examine the governance structure, objectives, and functions of JIHS and its influence on Japan’s public health governance. Eight key informants (A–H) were purposively selected based on: 1. involvement in JIHS policy, legislative, or organisational development; 2. documented roles in relevant government committees or JIHS governance bodies; and 3. ability to provide informed perspectives on institutional governance. One author of this report (TS) has extensive experience in Japan’s public health policy field and leveraged established professional relationships to identify and contact key stakeholders who met the inclusion criteria. Participants included one National Diet member (A) involved in legislative deliberations on JIHS establishment; four senior officials from the Ministry of Health, Labour and Welfare (MHLW) (B–E) with responsibilities spanning expert advisory coordination, infectious disease control policy, institutional integration planning, inter-ministerial coordination, and crisis management; and three JIHS Executive Officers (F–H) with expertise in global health, infectious disease research, and science–policy translation. Candidates were identified through public records, official JIHS leadership lists, and professional networks. Interviews were conducted in Japanese between August 4 and September 2, 2025, each lasting approximately 60 minutes, in person or online, using a semi-structured interview guide. All interviews were recorded with written informed consent, transcribed verbatim, and analysed in Japanese using Braun and Clarke’s [13] six-phase thematic analysis framework. Selected quotations were translated into English. We deliberately selected this framework rather than the 2021 Reflexive Thematic Analysis (RTA) because our study aimed to identify explicit, semantic-level patterns and required a systematic, replicable coding structure. The 2006 framework provides clear, stepwise procedures supporting consistency and transparency across multiple coders, whereas RTA emphasizes researcher subjectivity and interpretive reflexivity, which was less aligned with our descriptive analytical goals [14].

## Results/findings

### Key literature review

We reviewed five pieces of legislation, two grey literature documents, and eight government documents. Table 2 is a list of documents outlining the chronological progress towards the establishment of JIHS. It begins with the initial political recognition of the need for a Japanese version of the Centers for Disease Control (CDC) and progresses through a series of measures taken by the government and the Diet, culminating in the concretization of the national body’s philosophy, framework, and functions, supported by multiple legal bases, leading to JIHS’s establishment and official launch in 2025.

**Table 2:** List of key documents.

ID	Date	Title	Author	Document Type	Relevance to study
1	2020/2/17	The 201st National Diet, House of Representatives, Budget Committee [15]	House of Representatives, The National Diet of Japan	Government Document	Initial recognition of the necessity for establish Japan CDC
2	2020/6/26	Proposal for reform of national governance in the event of a large-scale infectious disease outbreak [2]	Working group, Liberal Democratic Party	Grey Literature	Reason of the necessity to create Japan CDC
3	2020/10/2	Recommendations from the Infectious Disease Control Governance Subcommittee of the Novel Coronavirus-Related Pneumonia Countermeasures Headquarters [3]	Liberal Democratic Party Policy Research Council	Grey Literature	Reason of the necessity to create Japan CDC
4	2022/6/15	Expert Council on COVID-19 Response. Mid- to long-term challenges for the next infectious disease crisis, based on past efforts against COVID-19 [16]	Expert Council on COVID-19 Response	Government Document	Reason of the necessity to create Japan CDC
5	2022/6/17	93rd Meeting of the Novel Coronavirus Response Headquarters [17]	Prime Minister’s Office of Japan	Government Document	Announcement to create Japan CDC
6	2022/9/2	Specific measures to prepare for the next infectious disease crisis based on past efforts regarding COVID-19 [18]	Social Security Council, Ministry of Health, Labour and Welfare	Government Document	Description on the specific needs of JIHS
7	2023/5/31	Act on Japan Institute for Health Security [19]	House of Councilors, The National Diet of Japan	Legislation	Legal foundation for JIHS establishment
8	2023/5/31	Law Concerning the Preparation of Related Laws in accordance with the Enforcement of the Act on Japan Institute for Health Security [20]	House of Councilors, The National Diet of Japan	Legislation	Legal foundation for JIHS establishment
9	2023/12/22	T-VISION [21]	Ministry of Health, Labour and Welfare	Government Document	Description on the specific needs of JIHS
10	2024/4/9	Towards the establishment of the National Institute for Health Risk Management and Research -Towards a society free from fear of infectious diseases- [22]	National Institute for Health Risk Management Preparation Committee	Government Document	Description on the specific needs of JIHS
11	2024/7/2	The National Action Plan for Novel Influenza, etc. [23]	The Cabinet Agency for Infectious Disease Crisis Management	Government Document	Description on the specific needs of JIHS
12	2025/4/1	Japan Institute for Health Security Mid-term goals [24]	Ministry of Health, Labour and Welfare	Government Document	Official launch and strategic goals
13	2025/4/1	Act on the Prevention of Infectious Diseases and Medical Care for Patients with Infectious Diseases [25]	The National Diet of Japan	Legislation	Official launch with legal foundation
14	2025/4/1	Act on Special Measures for Pandemic Influenza and Other Infectious Diseases [26]	The National Diet of Japan	Legislation	Official launch with legal foundation
15	2025/4/1	Community Health Act [27]	The National Diet of Japan	Legislation	Official launch with legal foundation

From the reviewed documents, we tabulated the recognised challenges in public health institutions prior to the establishment of the “Act on Japan Institute for Health Security” [19] and the “Law Concerning the Preparation of Related Laws in accordance with the enactment of the Act on Japan Institute for Health Security” [20], (hereafter referred to as JIHS Act) on May 31 2023.

Table 3 outlines the political and legal developments leading to the JIHS Act. From early political recognition of systemic weaknesses to expert reviews and legislative action, successive initiatives culminated in May 2023 with the enactment of the JIHS Act, establishing Japan’s foundation for integrated infectious disease governance.

**Table 3:** Recognised challenges in public health institution.

February 2020 - May 2023 Political and Legal Momentum Leading to JIHS Act
**Political recognition of systemic gaps**
• February 2020: Diet members urge a science-driven crisis management body (Japan Center for Disease Control and Prevention (Japan CDC)) [15].
**Party-led reviews and recommendations**
• June & October 2020: the ruling party, Liberal Democratic Party (LDP), identified legal/operational gaps, frames infectious disease as national security; urged new legislation and expert agencies [2,3].
**Expert reviews**
• June 2022: Expert committee highlighted unclear decision-making processes, weak surveillance systems and delays in Research and Development (R&D) [16,17].
**Government decision and legal foundation**
• June 2022: PM Kishida announced plans to create Cabinet Agency for Infectious Diseases Crisis Management (CAICM) and “Japan CDC” (via National Institute of Infectious Diseases (NIID)+National Center for Global Health and Medicine (NCGM) merger) [17].
• September 2022: Steps and processes for establishing CAICM and Japan CDC are presented [18].
• March 31, 2023: Japan Institute for Health Security (JIHS) Act is passed, providing the legal foundation for the merger of NIID & NCGM [19,^20^].

Table 4 summarizes post-enactment developments from 2023 to 2025. Following the JIHS Act, successive policy documents refined its conceptual design, defining intelligence, R&D, and clinical network roles. The integration into a national health crisis management institute was formalized with the official launch of JIHS in April 2025, and on the same day, MHLW provided the medium-term strategic goals of JIHS.

**Table 4:** Identified roles of JIHS.

June 2023 – April 2025 Post-enactment Developments
**Conceptual and structural design**
• December 22, 2023: *T-VISION* [21] outlines 3 core roles for JIHS:
• Intelligence (Domestic/global risk assessment)
• R&D Excellence (Accelerating R&D in medical countermeasures)
• Clinical-Trial Network (Nationwide coordination)
• April 9, 2024: *Towards the Establishment of JIHS* [22]
• Documented **roles and organisation structure,** including a cross-sectional crisis management Directorate
**Policy integration and expanded functions**
• July 2, 2024: The revised National Action Plan for Novel Influenza, etc. [23] described the JIHS’s role
**Official launch and strategic goals**
• April 1, 2025: JIHS is officially launched; MHLW assigned mid-term goals [24] to guide JIHS’s operations

As part of this study’s objectives, one research goal was to identify the key motivations for Japan’s infectious disease governance reform that led to the establishment of JIHS.

In 2020, Japan’s ruling party, the Liberal Democratic Party (LDP) issued a report from a working group reviewing the nation’s response. The report identified several challenges that Japan faced, including: 1. a lack of a clear chain of command, 2. a shortage of personnel with expertise in infectious disease crisis management, 3. lack of information technology capacities, 4. ineffective risk communication, 5. lack of flexibility in the system’s operation during crises, 6. lack of targeted infection control measures at the epi-center, and 7. delays in research and development [2].

In order to fundamentally strengthen Japan’s infectious disease crisis management, the LDP working group called for a technical organisation with functions for clinical response, public health response, crisis response operations, and research and development functions to carry out and support activities in a seamless manner. These movements illustrate the political and technical drivers that informed the reform process. To meet these needs and to respond to future public health crises, the government decided to merge NIID and NCGM into a new institution, the JIHS, tasked with the above functions.

Based on the key literature reviewed, the merger aims to make several improvements. First, it seeks to strengthen the scientific foundation for infectious disease response by integrating national data systems, research from basic to clinical stages, and facilitating collaboration with experts to rapidly provide evidence-based insights to MHLW and CAICM. It will also enhance emergency response through specimen testing, expert team deployment, and continuous training for healthcare and public health professionals. In addition, it serves to advance international health cooperation by promoting Universal Health Coverage, fostering global partnerships and talent development, and building regional clinical trial networks to accelerate diagnostics and treatment during health crises. Finally, the merger seeks to ensure the continued delivery of advanced medical care and ongoing responsibilities of both institutions, including the operation of a comprehensive hospital capable of providing cutting-edge treatment [18].

The following section analysed what changed before the enactment of the JIHS Act and other related laws. Tables 5 and 6 show the status before and after the enactment of the JIHS Act. Table 5 focuses specifically on NIID because while NCGM was already operating as a semi-private organisation, NIID – a governmental organisation – transitioned into a semi-private organisation as a result of the merger. This transition represents a more fundamental legal transformation than NCGM’s integration, as NIID shifted from operating solely under direct ministerial authority—without explicit mention in relevant legislation—to functioning as a “Special Public Corporation” with a dedicated enabling act, an expanded statutory mandate, and formal legal recognition across multiple laws. Under the former structure, NIID’s roles and responsibilities were not explicitly defined in law; rather, it carried out functions delegated by MHLW through administrative notifications and ordinances. The establishment of JIHS fundamentally altered this arrangement by granting the institution direct legal standing, clarifying its governance structure, and embedding its functions within Japan’s statutory framework.

**Table 5:** Legal status of NIID before the merger.

Law dimension	Before enactment of JIHS Act (Former NIID)
Legal Status	**A testing and research laboratory of the MHLW.**
	Had limited scope and authority for crisis management.
Oversight and Governance	**Strong ministerial oversight**, with its legal mandate, budget, and strategic direction controlled by MHLW.
Scope of Functions	Epidemiological surveillance, reference laboratory, risk assessment, and limited advisory roles; not specifically mentioned in laws.
	No direct involvement in clinical services and clinical trials.
Infectious Diseases Control Act [25]	NIID was not explicitly mentioned in the Act. Only the responsibilities of MHLW were defined.
Act on Special Measures for Pandemic Influenza and Other Infectious Diseases [26]	NIID was not explicitly mentioned in the Act. Only the responsibilities of MHLW were defined.
Community Health Act [27]	NIID and municipal public health institutes were not specifically mentioned. Prefectural and municipal public health centers consulted NIID mainly for testing or technical advice. Cooperation was based on administrative notifications, with no legal obligation or reporting requirements.

**Table 6:** Legal status of JIHS after the JIHS Act.

Law dimension	After enactment of JIHS Act
Legal status	**Special Public Corporation** (*tokushu-hōjin*) with a dedicated enabling act. Granted broader legal mandate and crisis management authority.
JIHS Act (Oversight and governance)	**Managed by a Board of Directors**. MHLW retains oversight authority, including setting mid-term goals.
JIHS Act (Scope of functions)	**Expanded mandate** includes: surveillance and intelligence, medical countermeasures, Research and Development acceleration, includes integration of research-to-practice pipeline, highly-specialized clinical function, nationwide clinical-trial network, training, human resource development, and international health cooperation.
Infectious Diseases Control Act [25]	JIHS explicitly is **designated as a core organisation for infectious disease prevention and response.** MHLW may entrust public health tasks, including specimen collection and investigations to JIHS.
Act on Special Measures for Pandemic Influenza and Other Infectious Diseases [26]	**JIHS identified as a key institute for disseminating scientific knowledge**. Amendments clarify JIHS’s role to provide scientific advice to the MHLW and the CAICM.
Community Health Act [27]	Amendments formalize **mutual cooperation between JIHS and prefectural and municipal public health institutes**.
	Local Public Institutes are to provide information and pathogens to JIHS, and JIHS is to provide technical guidance, and support capacity-building, especially during crisis.

JIHS was established as a special public corporation under the JIHS Act, enacted in May 2023 and effective from 1 April 2025. A “special public corporation” in Japan is a government-established organisation that undertakes public or administrative functions. Accordingly, JIHS operates with institutional independence and flexibility, enabling collaboration with private entities while remaining fully funded and closely supervised by the government.

Following the enactment of the JIHS Act and consequential amendments to related legislation, including the Infectious Diseases Control Act [25], the Act on Special Measures for Pandemic Influenza and New Infectious Diseases Preparedness and Response [26] (Special Measure Act), and the Community Health Act [27], JIHS was formally incorporated into Japan’s statutory framework. These legal revisions explicitly defined the institute’s institutional role, governance structure, and responsibilities.

While JIHS is mandated to provide expert advice to MHLW and the Cabinet Agency for Infectious Disease Crisis Management (CAICM), it remains under the administrative supervision of MHLW, which sets its mid-term objectives. The accompanying legal reforms including the National Action Plan for Novel Influenza, as stipulated in the Special Measures Act, also sought to strengthen Japan’s overall infectious disease response capacity, enhancing the procurement of supplies and human resources, reinforcing local public health center systems and improving information infrastructure.

**Fig 2:**
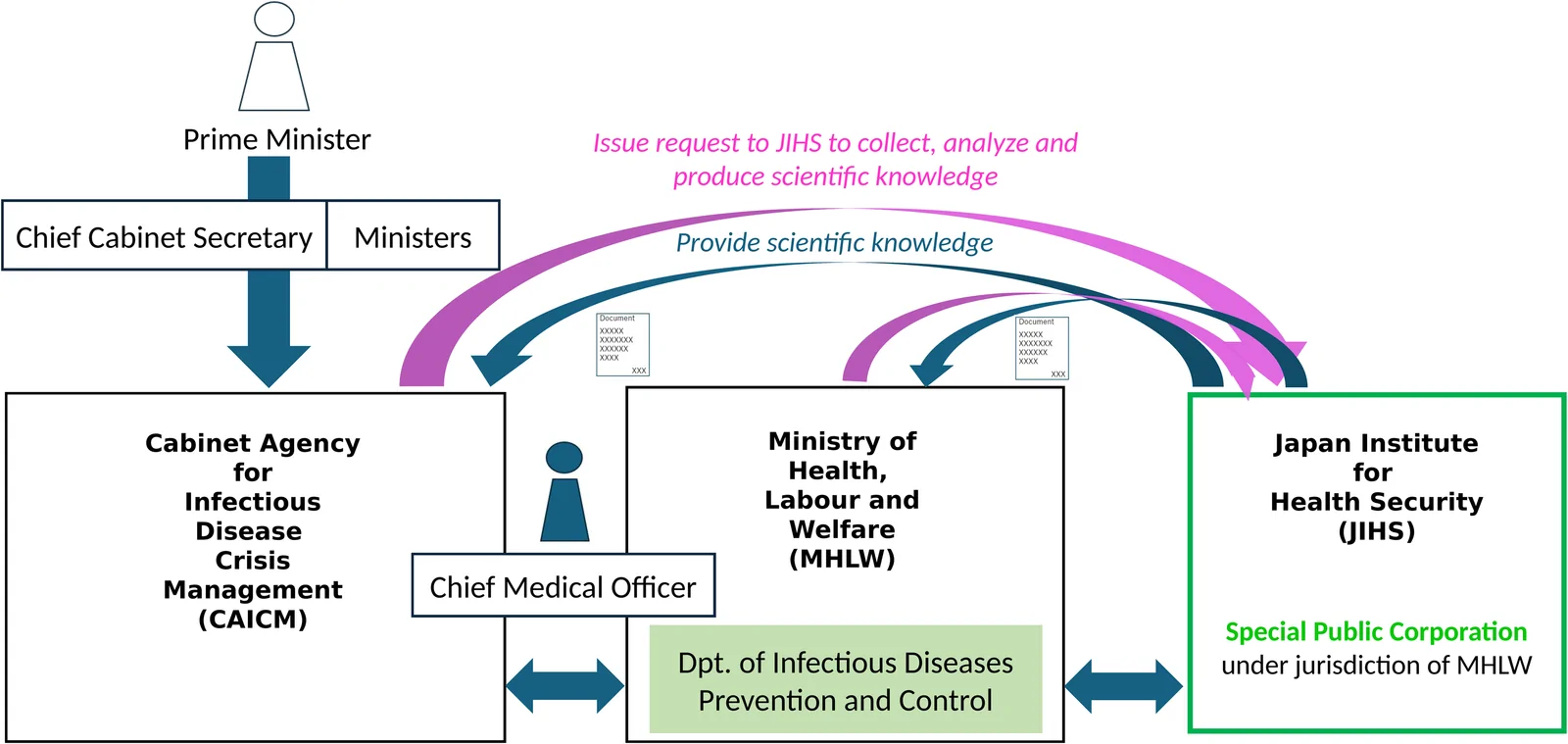
JIHS within the Government System. This figure was created by the authors. The governmental relationships depicted are based on publicly available information from the Cabinet Agency for Infectious Disease Crisis Management (CAICM) website (https://www.caicm.go.jp/en/about/index.html). All icons and graphical elements were newly created by the authors.

In 2023, to strengthen the government’s central command functions during health crises, Japan established CAICM through amendments to the Cabinet Act and the aforementioned Special Measures Act. To ensure coherence between scientific expertise and strategic decision-making, the MHLW’s Chief Medical Officer serves as a member of CAICM and acts as a liaison between the agency and MHLW. This arrangement reflects Japan’s broader effort to integrate scientific evidence into high-level policy coordination during public health emergencies. Fig 2 illustrates the institutional relationship between JIHS and its governmental counterparts, MHLW and CAICM.

These findings from the document review illustrate the institutional and legal transformation underlying the establishment of JIHS. To complement this documentary analysis, the following section presents insights from key informant interviews (KII) that clarify how these legal reforms were perceived and operationalized by policymakers and practitioners.

### Key informant interview

Based on the experiences and insights of the eight expert respondents in this study, the establishment of JIHS emerged from a complex interplay between political leadership, administrative coordination, and scientific expertise, revealing six interconnected themes that shaped this institutional transformation (Table 7).

**Table 7:** Summary of Key Themes, Representative Quotations, and Theoretical Implications.

Theme	Summary	Representative quotation (translated)	Theoretical implication
1. Legislative and Institutional Genesis	Political leadership initiated structural reform to overcome fragmented scientific advice during COVID-19. Bureaucrats mediated the technical drafting and coordination of the bill.	“There was no command structure capable of moving expertise and policy at the same time; we needed to create a Japan-style CDC.” (Interviewee A) “My role was to organise and provide necessary information for Diet deliberations.” (Interviewee H)	Political agenda-setting (policy window) and administrative feasibility as co-produced reform between politics and bureaucracy.
2. Governance Design	JIHS governance aimed to balance ministerial control (Cabinet Secretariat MHLW) and institutional autonomy through a dual-layer structure.	“We had to clarify who leads under normal and emergency conditions.” (Interviewee F) “Law alone cannot move people; motivation and a shared mission are essential.” (Interviewee H)	Balance of institutional authority and motivational legitimacy; the ‘governance paradox’ of hybrid public organisations.
3. Functional Integration	Integration of research, intelligence, and emergency functions required translation of abstract law into practical operations.	“Our job was to translate an abstract law into an operational system.” (Interviewee F) “Plans are ready, but developing a culture of implementation takes time.” (Interviewee H)	*Translation process* from design to practice; organisational learning as the foundation of capacity building.
4. Reconfiguration of Public Health Governance	JIHS introduced a more structured science–policy interface by formally connecting government, scientific expertise, and field-level implementation—a design intended to improve coordination across these three functions.	“A triangular structure connecting government, science, and the field has been established.” (Interviewee H) “Experts advise, but the final accountability lies with government.” (Interviewee C)	Boundary work in the science–policy interface; reconfiguration of epistemic legitimacy and accountability.
5. Organisational Culture and Human Agency	Legal merger completed, but cultural integration remains ongoing; leadership and shared mission are key to cohesion.	“Law can merge institutions, but not people’s mindsets overnight.” (Interviewee D) “It was the sense of mission, rather than external control, that motivated staff.” (Interviewee F)	*Embedded agency* through leadership and cultural learning; mutual shaping of institutional form and organisational ethos.
6. Future Outlook	JIHS’s establishment is viewed as a starting point; continuous learning and adaptation are required.	“The system itself must be refined through practice, not only through formality.” (Interviewee B) “The Japan model, which articulates research, medicine, and administration through coordinated but distinct roles, has global significance.” (Interviewee E, Interviewee G)	*Adaptive governance* and institutional learning; evolution of a Japan-specific model of embedded expertise.

### Theme 1. Legislative and Institutional Genesis: Political Leadership and policy movement

The journey began with a fundamental recognition: Japan’s fragmented response to COVID-19 exposed a critical gap in the command structure. As one senior politician reflected, “there was no command structure capable of moving expertise and policy at the same time; we needed to create a Japan-style CDC” (Interviewee A). This political vision, however, required careful translation into administrative reality. Government officials found themselves in a bridging role, as one explained: “My role was to organise and provide necessary information for Diet deliberations” (Interviewee H). The legislative process thus represented a negotiated compromise between political urgency for rapid reform and bureaucratic prudence in institutional design.

### Theme 2. Governance Design: Balancing Control and Autonomy

The governance architecture of JIHS reflected a delicate balancing act. The challenge was not simply creating a new organisation, but determining “who leads under normal and emergency conditions” (Interviewee F). The resulting structure established ministerial control through the Cabinet Secretariat and MHLW, while maintaining institutional autonomy for scientific work—what one participant called “a dual-layer structure.” Yet this formal design confronted a deeper truth articulated by another official: “Law alone cannot move people; motivation and a shared mission are essential” (Interviewee H). This observation points to the inherently adaptive nature of implementation. Legal frameworks, by design, establish broad parameters; operational adequacy is achieved through continuous calibration at the level of subordinate regulations, guidelines, and institutional practice. This experience reveals a key finding in legal preparedness scholarship: effective implementation requires that subordinate regulations, such as operational guidelines established under an overarching legal framework, be designed not only with technical authority in mind, but also with due consideration for the buy-in, values, and operational realities of those responsible for implementation.

### Theme 3. Functional Integration: Research, Intelligence, and Emergency Operations

The third theme captured the difficult transition from legislative text to functioning institution. As one respondent emphasized, “our job was to translate an abstract law into an operational system” (Interviewee F). This involved integrating the National Institute of Infectious Diseases’ research capabilities with the National Center for Global Health and Medicine’s clinical capacity, while incorporating disaster medicine units like Japan Disaster Medical Assistance Team (DMAT). However, the gap between planning and practice remained significant. One interviewee cautioned: “Plans are ready, but developing a culture of implementation takes time” (Interviewee H). This highlighted how institutional capacity depends not merely on legal authority but on embedded organisational learning and practiced routines.

### Theme 4. Reconfiguration of Public Health Governance

JIHS fundamentally reconfigured Japan’s public health governance by establishing what interviewees described as “a triangular structure connecting government, science, and the field “ (Interviewee H). This new architecture clarified roles: as one government official stated, “experts advise, but the final accountability lies with government” (Interviewee C). Yet this boundary proved more porous in practice than in theory. Under crisis conditions, multiple respondents noted, the distinction between scientific advice and political decision-making easily blurs, requiring continuous negotiation and trust-building among actors. Moreover, several interviewees emphasized that effective knowledge incorporation must extend beyond central institutions to include field-level actors—local health centers, prefectural public health institutes, and regional hospitals. One respondent characterized the ideal structure as a “triangle” connecting government policy, JIHS science, and frontline practice, while another stressed that regional networks proved indispensable during the pandemic response. However, interviewees acknowledged that such bidirectional knowledge exchange remains aspirational rather than fully realized, with the challenge lying in translating formal coordination mechanisms into genuine collaborative relationships built on trust. The reform thus reshaped not only institutions but also the epistemic relationships through which knowledge is authorized and legitimized in policymaking, though the incorporation of community-level knowledge remains an ongoing project.

### Theme 5. Organisational Culture and Human Agency

Perhaps the most persistent theme across interviews was the recognition that legal merger does not guarantee organisational integration. As one senior official bluntly observed, “law can merge institutions, but not people’s mindsets overnight” (Interviewee D). Another added: “It was the sense of mission, rather than external control, that motivated staff” (Interviewee F). This emphasized that institutional transformation is fundamentally a cultural project requiring leadership development, dialogue, and the cultivation of shared mission. The technical and logistical aspects of organisational design—reporting lines, legal authorities, budgets—proved insufficient without attention to the affective and ethical dimensions that sustain organisational commitment.

### Theme 6. Future Outlook: Learning and Adaptation

Finally, all interviewees emphasized that JIHS’s establishment marks only a beginning. As one stated, “The system itself must be refined through practice, not only through formality” (Interviewee B). The vision of integrating research, medicine, and administration into a distinctively Japanese model of health security requires sustained effort. Both interviewees noted the global dimension: “The Japan model, which articulates research, medicine, and administration through coordinated but distinct roles, has global significance” (Interviewee E, Interviewee G). Yet realizing these potential demands what participants described as continuous adaptation, recurrent training, and cultivation of reflective practitioners who can navigate uncertainty.

## Discussion and conclusions

Political awareness of Japan’s health system constraints was pointed out in Diet deliberations early in 2020, and after several stages, including the former Prime Minister announcing plans to establish a “Japan CDC” in 2022, the JIHS Act was passed in May 2023. After the adoption of the JIHS Act, various key public documents outlined the specific roles of the JIHS. The previous Minister of Health, Labour and Welfare outlined three core roles: intelligence, R&D excellence, and clinical trial network. In addition, in April 2024, the National Action Plan for Novel Influenza was fully revised for the first time since 2013, and MHLW set the JIHS’s mid-term goals on the official launch date of JIHS [24]. In conjunction with the establishment of the JIHS Act, the Infectious Diseases Control Act, the Special Measures Act, and the Community Health Act were amended to provide the overall legal basis necessary for JIHS to fulfil its intended role. Thus, the framework was instituted to delineate the organisational structure and functional responsibilities of JIHS. In particular, the JIHS Act outlines its governance, structure, and role, and the Special Measure Act, Infectious Diseases Control Act, and Community Health Act articulate JIHS’s responsibilities. In accordance with the JIHS Act, JIHS is required to formulate mid-term goals, a mid-term plan, and an annual plan. Furthermore, the National Action Plan for Novel Influenza, as stipulated in the Special Measures Act, obligates JIHS to develop a Pandemic Business Plan.

This study demonstrates that the establishment of JIHS represents one of the transformations of Japan’s infectious disease governance system. The integration of NIID and NCGM under a new legal and organisational framework has created a Special Public Corporation designed to bridge scientific research and clinical practice, and scientific evidence to policy made in government. The JIHS Act and associated legal reforms have clarified institutional mandates, introduced new mechanisms for coordination across ministries and local governments, and embedded preparedness and R&D capacity as core elements of national health security.

The findings from document analysis and KII consistently indicate that the legal foundation of JIHS enhances both vertical and horizontal coordination within Japan’s health system. The shift from a ministerial institute to a semi-autonomous public corporation provides operational flexibility for research collaboration while maintaining strong oversight by MHLW. This institutional reconfiguration is consistent with the OECD’s observation that effective governance requires a balance between operational discretion and accountability mechanisms, avoiding both excessive control and insufficient governance [28]. In this sense, the transition to a semi-autonomous public corporation can be understood as a concrete manifestation of such governance principles.

KIIs illuminate the interactional governance among politicians, government officials, and experts during the institutionalization of crisis management in Japan. Four interconnected dynamics emerged from the analysis: political steering, administrative mediation, science-policy interface negotiation, and cultural-institutional co-production. Rather than operating as discrete phases, these dynamics functioned as recursive, mutually constitutive processes characteristic of collaborative governance systems [29].

### From crisis window to institutional innovation

The COVID-19 pandemic opened what Kingdon [30] terms a “policy window,” enabling political actors to overcome institutional inertia that had previously blocked comprehensive reform. Political leadership provided normative direction and leveraged crisis momentum to legitimize the establishment of a Japan-style CDC. However, translating political intent into operational reality required extensive administrative mediation. Bureaucrats functioned not merely as technical implementers but as institutional entrepreneurs, navigating between political urgency, legal constraints, and organisational capacity. This mediating role reveals a critical lesson in legal preparedness: while legal frameworks establish the foundational mandates, authorities, and accountability structures necessary for effective governance, their translation into practice depends on the development of subordinate instruments—such as guidelines, operational protocols, and inter-agency agreements—that reflect the mission, culture, and values of those responsible for implementation. The administrative challenge encountered at JIHS, where bureaucrats and JIHS leadership together were required to cultivate organisational commitment after the legal framework had been established, suggests that these subordinate instruments were not sufficiently developed in parallel with the legal reform process. One official noted, “law alone cannot move people; motivation and a shared mission are essential” (Interviewee H). Such findings align with neo-Weberian perspectives on administrative rationality [31] while highlighting the relational dimensions that formal institutional analysis often overlooks.

### Restructuring the science-policy interface

The JIHS reform fundamentally reconfigured how scientific expertise relates to policy authority. The new structure clarified that experts of JIHS advise while final accountability lies with government, institutionalizing a distinction between scientific judgment and political decision-making. Yet this boundary proved more porous in practice than in theory. Under crisis conditions, interviewees noted, the distinction easily blurs, requiring what Ansell and Gash [32] characterize as “face-to-face dialogue” and “trust-building” among collaborative partners. Experts thus function as epistemic brokers [33], translating complex scientific knowledge into policy-relevant information, but their influence depends on relational trust and institutional positioning rather than autonomous authority. The findings indicate that the establishment of JIHS has implications not only for institutional coordination but also for how knowledge is structured and mobilized within Japan’s public health governance. Although recent legal reforms formally clarify the distinction between scientific advice and political accountability, interview data suggest that effective governance depends on directional and bidirectional knowledge flows across levels. Rather than a unidirectional transfer of expertise from central institutions, respondents described an emerging triangular configuration linking government decision-making, scientific expertise within JIHS, and field-level practice in local and prefectural public health institutions. However, this configuration remains only partially realized in practice, as its functioning relies heavily on relational trust, shared professional norms, and informal communication developed through experience. Accordingly, the reconfiguration of the science–policy interface through JIHS should be understood as an ongoing process of epistemic and organisational alignment rather than a completed institutional design.

### Cultural-institutional Co-production

Perhaps the most significant finding concerns the cultural dimensions of institutional reform. The formal legal merger of NIID and NCGM created unified organisational structure, but actual integration required transforming organisational identities, values, and practices. As one interviewee observed, “organisational culture transformation requires 10 years” (Interviewee D), while another emphasized that JIHS establishment is “not just creating the box but putting soul into it” (Interviewee E). This insight resonates with Lawrence and Suddaby’s [34] concept of “institutional work”—the purposive action through which actors create, maintain, and transform institutions. Formal rules provide the framework, but organisational culture gives them meaning and motivational force. This co-production process operates through leadership vision, professional socialization, performance evaluation, and everyday practices that gradually align individual identities with organisational mission.

### Toward a Co-production Model of Crisis Governance

Taken together, these findings reveal a governance model built on ongoing collaboration rather than fixed chains of command. In this model, scientific knowledge, administrative design, and political decision-making do not operate in isolation or in a strict top-down sequence; instead, they continuously inform and adjust one another over time. JIHS embodies this approach: its institutional legitimacy rests not on any single source of authority, but on the combined contributions of political accountability, administrative capacity, and scientific expertise working in concert. This tripartite structure reflects what Emerson et al. [29] term “principled engagement”—a collaborative dynamic sustained through ongoing discovery, definition, deliberation, and determination among interdependent actors. Japan’s experience thus contributes to comparative governance theory by demonstrating how pandemic crisis catalysed a new mode of embedded expertise: an institutional attempt to internalize scientific advice within the state apparatus while preserving the autonomy and trust upon which scientific credibility depends. The durability of this innovation, in the manner of restructuring, will depend on sustained investment in the relational infrastructure—shared understanding, mutual trust, and collaborative capacity—that underpins effective crisis governance.

The objective of our study was to understand the new governance arrangements for, and objectives of, Japan’s health security system, which includes, clarifying the goals of reforming Japan’s health security system, understanding the governance and functions of JIHS to meet the goals, and identifying the reforms conducted, including new laws and amendments to existing laws, to establish JIHS. The results from both the document review and interviews confirmed that the necessary legal frameworks have been implemented, representing a foundational step toward strengthening Japan’s health security system. Effective implementation of these frameworks, however, depends on the concurrent development of subordinate instruments that translate legal mandates into practice in a manner that reflects the operational realities and institutional cultures of those responsible for implementation.

Another interpretation suggests that while legal clarity has improved, actual governance practices may remain dependent on political dynamics. Decision-making rules exist in formal documents, yet their institutional penetration and staff awareness are incomplete. Additionally, there are challenges in risk communication and the absence of direct communication authority over citizens. Although relevant regulations and official guidelines describe frameworks for risk communication and public information dissemination, the actual effectiveness and operational feasibility of these mechanisms remain unclear in practice when implemented during real crises, which would need to be carefully revisited by the Japanese government, and assessed for a more effective approach.

The study’s conclusions are internally valid to the population directly involved in the establishment and operation of JIHS. The combination of legal document review and semi-structured interviews with policymakers, civil servants, and institutional leaders provides a robust and triangulated understanding of the governance reform process.

The experience of JIHS offers important but context-dependent lessons for other countries seeking to strengthen their public health governance. The legal design of a Special Public Corporation is uniquely suited to Japan’s administrative tradition, where autonomy is balanced with ministerial control. Consequently, while the general principles, such as integrating research, policy, and clinical capacity, are transferable, the precise institutional model may not be universally applicable.

Comparatively, transformations in other jurisdictions in many other countries were driven by analogous crises but embedded in different political-administrative systems [35]. JIHS case therefore underscores the importance of tailoring governance reforms to national legal and bureaucratic contexts. Rather than offering a directly transferable institutional blueprint, this case study provides generalizable insights into governance processes—such as the role of legal design in enabling coordination, the management of science–policy boundaries, and the importance of institutional learning—that are likely to be relevant across diverse health system contexts. Future evaluations of JIHS’s performance will be essential to determine whether its legal framework can deliver the intended improvements in pandemic preparedness and response.

### Limitations

Given that the interviews primarily involved individuals closely engaged with and directly involved in JIHS’s establishment, the findings should be interpreted as reflecting the perspectives of actors most directly involved in policy design and institutional formation. Consequently, the perspectives captured in this study reflect how policymakers and institutional designers understood the reform in terms of intended function and governance, rather than how it has been experienced by those responsible for operational implementation. While this insider focus provides valuable insight into the policy rationale and legal reasoning that underpinned the reform process, the findings should not be generalized to represent the full spectrum of stakeholder perspectives. Nevertheless, they offer a coherent and empirically grounded account of the institutional logic and governance intentions that shaped the establishment of JIHS.

### Policy recommendations

The findings of this study demonstrate that the establishment of JIHS emerged through an interactional governance process involving political actors, bureaucrats, and experts. Across themes 1–6, a consistent pattern emerged: Japan’s legal framework for pandemic response was significantly reformed, yet the operational adequacy of that framework—as realized through subordinate legislation, institutional arrangements, and front-line practice—has not been systematically validated. This gap, between statutory design and demonstrated operational performance, represents an issue that warrants continued examination in national preparedness, through the sub-statutory and operational layers at which effective implementation is ultimately determined.

Drawing on the six themes, which emphasize political agenda-setting (Theme 1), administrative design and implementation (Themes 2–3), the science–policy interface (Theme 4), organisational culture and human agency (Theme 5), and continuous institutional learning (Theme 6), we propose a governance improvement model that connects legislative authority with practical functionality in emergency conditions.

1. *Strengthening the Legal Foundations Through Systematic Assessment (Themes 1–2)*

The Findings highlighted that political leadership provided the momentum for legal reform (Theme 1), and bureaucrats translated this reform into legislative and administrative design (Theme 2). However, despite these achievements, there has been no comprehensive public assessment of whether existing legal authorities are adequate or sufficiently clear during pandemic emergencies.

A systematic evaluation mechanism is therefore necessary to understand how the legal framework functions under real crisis pressures. That said, a systematic evaluation mechanism of the kind is already called for under Level 5 of Indicator P1 in the WHO JEE Tool 3.0. Such an assessment would ensure alignment between political intent, administrative feasibility, and the operational realities that emerge in emergency governance.

2. *A Multi-Stakeholder, Inter-Agency Evaluation Model for Legal Authorities (Themes 2–3)*

Themes 2 and 3 revealed that bureaucrats play a mediating role by transforming abstract legal provisions into operational systems. However, while bureaucrats were engaged in the implementation process, their involvement remained primarily at the administrative and coordinative level rather than at the technical and operational level where day-to-day implementation decisions are made. This limited depth of engagement helps explain the gaps that remain between legislative design and practical implementation, as the perspectives and expertise of technical-level implementers were not sufficiently reflected in the subordinate instruments, such as operational protocols and inter-agency agreements, that are necessary to translate legal mandates into operational practice. Building on this insight, we recommend developing a collaborative evaluation mechanism led jointly by JIHS, MHLW, and CAICM.

This mechanism would: assess the applicability of legal authorities during simulated emergency conditions, identify procedural, jurisdictional, or coordination challenges, test command structures and communication pathways, and reveal inconsistencies between formal rules and operational realities.

This approach operationalizes the administrative mediation observed in Themes 2–3 and strengthens the organisational learning necessary for capacity building.

3. *Evaluating the Science - Policy Interface and Decision-Making Boundaries (Theme 4)*

Theme 4 identified the creation of a “triangular structure” connecting government, science, and local implementation, while also demonstrating that boundaries between expertise and political judgment blur during crises.

The proposed assessment framework should therefore examine: how scientific advice is generated, interpreted, and embedded within administrative decision-making; the transparency and timing of expert input; the degree to which expert recommendations inform political decisions; and the practical feasibility of risk communication pathways, particularly given the lack of direct authority over public communication noted by some informants.

This would ensure that the reconfigured science-policy interface functions effectively under emergency conditions rather than only in formal documents.

4. *IncorporatingOrganisational Culture and Human Agency into Legal Evaluation (Theme 5)*

Theme 5 emphasized that legal mergers do not automatically produce cultural integration. Staff motivation, shared mission, and leadership are essential drivers of organisational effectiveness.

Thus, evaluations must extend beyond formal authority and include: the extent to which staff across JIHS share a common operational culture; capacity for cross-agency collaboration; trust, communication norms, and readiness for joint action; alignment between organisational identity and legal mandates.

These factors reflect the cultural-institutional co-production required for reforms to function as intended.

5. *Institutionalizing a Continuous Learning and Adaptation Cycle (Theme 6)*

Theme 6 stressed that the establishment of JIHS is “only the beginning” and that the system must be refined through practice. Consistent with the “legal surveillance” component of legal preparedness as defined by the GHSA LP AP, we recommend institutionalizing a cyclical process of testing, evaluation, feedback, and reform to ensure the iterative review and adaptation of legal frameworks over time. This iterative mechanism would: reveal gaps between law and practice, enable adaptive governance across agencies, cultivate reflective practitioners, and ensure that Japan’s hybrid model of health security evolves in response to experience.

This approach aligns with the concept of learning health system, defined by Sheikh and Abimbola as one that embeds continuous learning and adaptation into routine governance structures, moving beyond static preparedness frameworks by embedding continuous learning and adaptation into routine governance structures [36]. By institutionalizing such ongoing evaluation, JIHS can progress from establishing statutory framework to achieving operational adequacy that is continuously tested, validated, and strengthened through the subordinate regulations, institutional arrangements, and operational guidance that give practical effect to the law.

### Expected outcomes and international relevance

Implementing this legal assessment process, consistent with the legal assessment component of legal preparedness as defined by the GHSA LP AP, would yield several benefits: enhanced transparency in assessing the adequacy of legal authorities; improved understanding of inter-agency coordination under crisis conditions; evidence-informed adjustments to Japan’s pandemic response laws; strengthened coordination and alignment of research, medicine, and administration in the “Japan model.”

Although rooted in Japan’s hybrid governance culture, balancing autonomy and ministerial oversight, the model can be adapted for use in other national systems, with adjustments for local legal and administrative traditions.

In sum, the policy recommendations presented here translate the six themes into a coherent, practice-oriented model for strengthening Japan’s pandemic preparedness. By connecting political agenda-setting (Theme 1), administrative design and implementation (Themes 2–3), science–policy boundary work (Theme 4), organisational culture (Theme 5), and adaptive governance (Theme 6), the proposed evaluation framework provides a sustainable mechanism for ensuring that legal reforms are not only enacted but also functional under real-world emergency conditions.

## Conclusions

This study examined the legal and institutional reform process that led to the establishment of JIHS as a new governance model for infectious disease preparedness and response. By analyzing legislative developments and key informant perspectives, we found that the JIHS Act and related amendments clarified institutional mandates, enhanced coordination across government and expert bodies, and embedded research, clinical, and policy integration within Japan’s public health system. Before the Act was enacted, policy challenges were comprehensively mapped through deliberations in government expert advisory councils, providing a foundation for ex ante evaluation. Consistent with the nature of Japanese framework legislation, the JIHS Act delineates broad institutional parameters, with operational adequacy subject to continuous refinement through subordinate regulations and administrative implementation. However, our findings also indicate that operational adequacy depends critically on the quality of their design process, particularly the extent to which technical-level implementers are meaningfully consulted from the outset, and on the establishment of ongoing mechanisms to test, validate, and adapt frameworks through practice. The central question, therefore, is whether the established framework functions effectively in practice, an implementation-phase inquiry in which operational adequacy continues to be calibrated through sub-statutory layers.

To address this, we proposed a multi-phase, multi-stakeholder exercise framework that links legal design to operational performance, which aligns with the legal assessment and legal surveillance elements of the GHSA LP AP framework and consistent with WHO JEE Tool 3.0 standards. This framework not only reflects Japan’s unique hybrid governance structure but also provides a transferable model for evaluating and strengthening pandemic preparedness in other countries. Ultimately, this study highlights that sustainable health security depends on continuous institutional learning—where law, science, and governance evolve together to ensure agility and public trust in future health emergencies.

## Abbreviations

CAICM: Cabinet Agency for Infectious Disease Crisis Management
CDC: Centers for Disease Control and Prevention
COVID-19: Coronavirus Disease 2019
DMAT: Disaster Medical Assistance Team
JIHS: Japan Institute for Health Security
LDP: Liberal Democratic Party
MCM: Medical Countermeasure
MHLW: Ministry of Health, Labour and Welfare
NCGM: National Center for Global Health and Medicine
NIID: National Institute of Infectious Diseases
R&D: Research and Development
WHO: World Health Organization
