# Supplementary material for: Legal design for establishing a novel parastatal public health institute – Japan Institute for Health Security: A case study
Source: PLOS Glob Public Health. 2026 Aug 26;6(8):e0007088. doi: 10.1371/journal.pgph.0007088 (PMC13514060; doi:10.1371/journal.pgph.0007088)
Supplement: Supplementary file 2 [file pgph.0007088.s003.docx]

**ANNEX**

The individual comments and our responses are provided in the table below. And each comment is as follows.

| **Line #** | **Comment** | **Response** |
| --- | --- | --- |
| 41-43  P6-C1 | Although this might be the case as a finding, noting that this framing raises concerns about the message it sends on legal preparedness. Informants seem to misunderstand how legal frameworks should be designed under a legal preparedness approach. Legal frameworks provide rules, including roles and responsibilities and accountability mechanisms, to guide implementation. A different framing would be more appropriate (such as "Informants confirmed the importance of ensuring that interagency trust and cultural integration between merged institutions is critical to the effective implementation of legal frameworks" (omitting the "however" and "insufficient for operationalization)) | We agree and have revised this sentence. The sentence has been reframed to remove the suggestion that legal frameworks are insufficient. The revised text now reads: "Informants confirmed the importance of ensuring that inter-agency trust and cultural integration between merged institutions for the effective implementation of legal frameworks," omitting the original "however" and "insufficient for operationalization.” |
| 48-49  P6-C2 | This conclusion is concerning. Risks undermining the value of legal frameworks in pandemic preparedness and response, particularly for the less versed. This assumes that the JIHS Act was well designed, but based on what has been described here, there are major gaps that make it seem like it was not sufficiently thought out and that keep it from being effective. For a legal framework to "determine" whether legal frameworks deliver intended improvements in pandemic preparedness and response, it would have to be well designed. It is dangerous to claim that the JIHS Act to represents all legal frameworks. Strongly recommend familiarizing with legal preparedness concepts, including the GHSA Legal Preparedness Action Package definition of "legal preparedness" in the context of public health emergencies, the JEE Tool 3.0 "Legal Instruments" technical area, "Legal Preparedness as a Foundation for Global Health Security" (2025) (https://pubmed.ncbi.nlm.nih.gov/40485256/) | We thank the reviewer for this crucial point. The sentence has been revised to clarify that the evaluation pertains specifically to the design choices of the JIHS Act, and references to legal assessment and surveillance mechanisms under the GHSA LP AP(<https://globalhealthsecurityagenda.org/wp-content/uploads/2024/08/Final_May2023_Legal-Preparedness-Definition.pdf>, https://www.elgaronline.com/view/journals/jghl/1/1/article-p88.xml) and WHO JEE 3.0(https://iris.who.int/server/api/core/bitstreams/f9a1d72f-d8e4-4abe-900f-040829de686d/content) have been added. |
| 121-122  P10-C1 | Strongly recommend expanding on the definition of legal preparedness and its five core elements, per the GHSA Legal Preparedness Action Package, before delving into discussing the adequacy of the JIHS Act. It will help frame the discussion/analysis. | We thank the reviewer for this important point. A new paragraph has been added defining legal preparedness per the GHSA LP AP, including its five core elements, and referencing the WHO JEE 3.0 Legal Instruments technical area. This framing is now used throughout the analysis. |
| 122-123  P10-C2 | Assuming that there are no records of Japan's assessment before the development of the JIHS Act. Of note, an effective legal framework, to be effective, should take into account all findings from the assessment of gaps in implementation (legal authorities needed, for example). This is the only way that a legal framework can support effective implementation. This assessment cannot happen after the legal framework has been developed/after the fact. | We thank the reviewer for this important observation, and we fully agree that a legal framework should be informed by a prior assessment that identifies the problems it is intended to address. We recognize that our original wording could be read as implying that the JIHS Act was developed without such an assessment, and we have revised the text to correct this impression.  In fact, the establishment of JIHS was preceded by government review processes in 2022 that systematically identified the institutional and legal shortcomings exposed by Japan's COVID-19 response. The Expert Meeting on the Response to COVID-19 (June 2022) noted deficiencies in the decision-making process and in the adequacy of science-based assessment, and the subsequent decision of the Government COVID-19 Response Headquarters (September 2022) explicitly identified the dysfunction of the central command function, sectoral silos among relevant agencies, and inadequate coordination between national and local governments. The JIHS Act was developed on the basis of these findings. We have revised the manuscript (lines 150–157) to make this sequence explicit.  It is also important to note a structural feature of the Japanese legislative system. Statutes enacted by the Diet establish the basic framework, authorities, and principles, while the operational details are delegated to subordinate legislation (cabinet and ministerial ordinances, notifications) and to internal regulations and operational guidance. The Cabinet Legislation Bureau review ensures the legal coherence of this framework, and in this respect the JIHS Act is soundly designed. Accordingly, the design challenge we identify does not concern legal-technical deficiencies in the Act itself, but rather the layer at which operational adequacy is determined in the Japanese system—namely, the subordinate regulations and operational arrangements that give effect to the statutory framework. It is at this level that the operational realities and institutional culture understood by front-line implementers must be reflected, and we now position the limited reflection of this perspective as a central lesson of the JIHS case.  While the problems were identified and translated into legislation, whether the reformed framework proves operational and effective in practice has not yet been tested. We have reframed the relevant passages so that the future evaluation we describe refers specifically to the real-world operationalization of an already-enacted framework—consistent with the legal surveillance element of legal preparedness (GHSA LP AP; Negri et al., 2024)—rather than to the adequacy of the legislative process itself. |
| 129  P11-C1 | Recommend adding: "both in Japan and globally" for clarity and emphasis on the contribution in and beyond Japan. | Accepted. The phrase "both in Japan and globally" has been incorporated to clarify the broader relevance of the contribution. |
| 136  P11-C2 | Appears to be a "typo." "Diet" is capitalize in all other instances. | Accepted. The capitalization of "Diet" has been corrected for consistency. |
| 173  P13-C1 | Noting that the very tracking of the various steps that it took to establish the JIH is itself a contribution to the world, particularly for countries who are looking to do the same. | We appreciate the reviewer’s comment. The chronological documentation of the steps leading to the establishment of JIHS is retained as a contribution to international literature. |
| 208  P16-C1 | It is unclear what "these findings" is referring to (the findings in the previous paragraph?). Recommend clarifying. | We appreciate the reviewer’s comment. "These findings" has been replaced with "These movements" to clarify the referent. |
| 220  P16-C2 | Typo. Missing a space. | Accepted. The typographical error (missing space in "toensure") has been corrected. |
| 223  P17-C1 | Grammar. Remove. | Accepted. The extraneous word "has" has been removed. |
| 227  P17-C2 | Recommend elaborating more on why | We appreciate the reviewer’s comment. The passage explaining NIID's legal transformation has been expanded. The revised text clarifies the fundamental shift from operating under ministerial authority without explicit statutory mention to functioning as a Special Public Corporation with formal legal recognition. |
| 232  P17-C3 | Recommend reviewing formatting and punctuation in both tables for consistency in approach | We appreciate the reviewer’s comment. Formatting and punctuation in both tables have been reviewed and made consistent. |
| Table5  P17-C4 | For example, should this be period? | Accepted. Punctuation has been reviewed and corrected for consistency throughout the tables. |
| Table5  P18-C1 | another example: should this be a period? | Accepted. Punctuation reviewed and corrected. |
| Table6  P18-C2 | typo. Extra space. | Accepted. The extra space ("t a") has been corrected. |
| Table6  P18-C3 | period? | Accepted. Punctuation corrected. |
| Table6  P18-C4 | should this be a period? | Accepted. Punctuation reviewed and corrected. |
| 235  P18-C5 | Shouldn't this be "Act" (singular)? | Accepted. Corrected to "Act" (singular) for consistency. |
| 236  P18-C6 | Since defining the term in this sentence, recommend placing in quotations. | Accepted. "Special public corporation" has been placed in quotation marks as it is being defined in that sentence. |
| 247  P19-C1 | Delete to avoid using too many commas and help with flow | Accepted. "Which will be" has been deleted to improve flow and reduce unnecessary comma usage. |
| 283-285  P20-C1 | Caution to the authors. Using this quote to frame the argument posits that law is separate from motivation, culture, and mission, where "law" (at its basic) should reflect a society's values, principles, and commitments to guide behavior. Based on how legal preparedness has been understood and defined by experts, I would observe that the experience of Japan clearly shows the unintended and unfortunate consequences that results from designing a legal framework that is not informed by the mission, motivation, and mission from the start, which significantly comes from the input from technical-level experts who are most familiar the culture at the implementation level (this is critical to ensure that a legal framework response to reality). In other words, when designing legal frameworks, the shared mission, good practices, and lessons learned (as underwood by the implementers) should be front and center and from the very start. Otherwise, the implementation of the legal framework results in the JIHS experience as described by the authors. | We thank the reviewer for this crucial point. The passage has been substantially revised. The original framing, which used the quote "Law alone cannot move people" to suggest a tension between legal frameworks and motivation, has been reconsidered. Rather than positioning law and shared mission as separate or oppositional, the revised text clarifies that legal frameworks establish broad parameters, with operational adequacy achieved through continuous calibration at subordinate levels. The passage now explicitly acknowledges a key insight from legal preparedness scholarship: that effective implementation requires subordinate regulations and operational guidelines to be designed with due consideration for the values, buy-in, and operational realities of those responsible for implementation. This reframing better reflects the reviewers' observation that shared mission and lessons learned from implementers should inform legal framework design from the outset. |
| 285-286  P20-C2 | This analysis/point is unclear. Important to define what is meant by "motivational legitimacy." Also, if the statement is true, then there is a problem with the way that the JIHS was designed--its value is as an adequately designed legal framework is questionable. | We appreciate the reviewer’s comment. The phrase "motivational legitimacy" has been removed and the explanation merged to the previous sentences. |
| 294-296  P21-C1 | Yes, it's implementation supported by legal frameworks. Consider: legal frameworks can help expedite implementation if their design is meaningfully informed by practioners/implementers. (not imposed on, such as by politicals) | We appreciate the reviewer’s comment. The text has been revised to reflect that legal frameworks can support and expedite implementation when their design meaningfully incorporates input from practitioners and implementers. |
| 300-302  P21-C2 | This is good to know | We appreciate the reviewer’s comment. The passage is retained as an informative finding on the defined boundary between scientific advice and governmental accountability. |
| 302-304  P21-C3 | Yes, it does. This is where clearly designed legal frameworks outlining all the roles and responsibilities are critical. | We appreciate the reviewer’s comment. The importance of clearly designed legal frameworks that outline roles and responsibilities is now explicitly noted in the revised discussion. |
| 309  P21-C4 | Yes, this is the nature of implementation (partnership building is a critical component) | We agree with the reviewer’s view. The point regarding the nature of implementation and the importance of partnership building is acknowledged and retained. |
| 320  P22-C1 | Yes, it is. Legal frameworks should be designed in a way that supports this transformation / cultural project. It is critical that they reflect the culture. The assessment of a legal framework needs to be iterative. | We appreciate the reviewer’s comment. The text has been revised to emphasize that legal frameworks should be designed iteratively and in a manner that reflects the culture and operational realities of those responsible for implementation. |
| 331-332  P23-C1 | Yes this reflects and aligns with the core components of "legal preparedness" as defined by the GHSA Legal Preparedness Action Package, namely: legal assessment, capacity, and implementation tools. | We appreciate the reviewer’s comment. The alignment with the GHSA LP AP's core components of legal preparedness—legal assessment, capacity, and implementation tools—has been made explicit in the revised text. |
| 342  P23-C2 | Shouldn't this be "JIHS Act" to be consistent with approach elsewhere (how the legal framework is referenced). | "Establishment of the JIHS" has been revised to "JIHS Act" for consistency with usage elsewhere in the manuscript. |
| 366-367  P24-C1 | Repetitive with earlier sentence | We appreciate the reviewer’s comment. The repetitive sentence has been removed. |
| 374  P24-C2 | Noting that these sub-headings should be formatted to stand out (bolded/italics/underline) and not seem like part of the text. | Accepted. Sub-headings have been reformatted (bolded/italicized) to clearly distinguish them from body text. |
| 381-383  P25-C1 | Again, this relies heavily on misunderstanding of legal preparedness concept and its goals/objectives and how it aims to support pandemic preparedness | We appreciate the reviewer’s comment. We agree and have revised this passage. The original framing used the quote "law alone cannot move people" to suggest that legal frameworks are inherently limited in motivating behavior, which the reviewer correctly identified as inconsistent with legal preparedness concepts. The revised text repositions this finding within a legal preparedness framework: rather than implying that law and motivation are separate, it now clarifies that legal frameworks establish foundational mandates, authorities, and accountability structures, and that their effective translation into practice depends on the concurrent development of subordinate instruments—such as guidelines, operational protocols, and inter-agency agreements—that reflect the mission, culture, and values of those responsible for implementation. |
| 388  P25-C2 | What experts are we talking about? Government experts, non-government experts? | We appreciate the reviewer’s comment. The term "experts" has been clarified to specify the types of experts being referenced in context. |
| 421-424  P27-C1 | This statement is hard to follow. I know that it introduces some more abstract concepts, but highly recommend revising with plain English to the extent possible. Otherwise, it's easy to get lost on the takeaway that the article is trying to convey here. Unclear why contrasting "recursively" with "hierarchical" (they are not mutually exclusive). if what is meant is an iterative process, "iterative" might be a better word than "recursive." | We appreciate the reviewer’s comment. The passage has been rewritten in plain English. "Recursively" has been replaced with a clearer explanation of ongoing, iterative collaboration among scientific knowledge, administrative design, and political decision-making. |
| 430  P27-C2 | what is this referring to? JIHS? Way of governance? | We appreciate the reviewer’s comment. "This innovation" has been revised to "in the manner of restructuring" to clarify the referent. |
| Table7  P28-C1 | What this experience and conclusions point to is a lack of legal preparedness expertise in Japan. There seems to be insufficient knowledge among government officials of the field. There could be important opportunities to strengthen capacity in this area for legal frameworks to meet reality and the needs. | We appreciate the reviewer’s comment. The table entry has been revised to more accurately reflect JIHS's design intent: formally connecting government, scientific expertise, and field-level implementation to improve coordination across these three functions. |
| 445-446  P28-C2 | Then the approach may not have been done adequately | We appreciate the reviewer’s comment. We agree and have revised this passage. The original framing, which positioned legal frameworks as insufficient and highlighted concerns about cultural integration as a separate limitation, has been reconsidered in light of the reviewer's observation that such concerns may reflect inadequacies in the implementation approach itself. The revised text removes the original "however" construction that implied legal frameworks alone were inadequate, and instead reframes the finding to emphasize that effective implementation depends on the concurrent development of subordinate instruments that translate legal mandates into practice in a manner that reflects the operational realities and institutional cultures of those responsible for implementation. |
| 453-455  P29-C1 | These probably need to be revisited by the Japanese government and assessed for a more effective approach. | We appreciate the reviewer’s comment. The revised text notes that risk communication frameworks would need to be carefully revisited by the Japanese government and assessed for a more effective approach. |
| 468  P29-C2 | Yes, this is the right question to ask given the outcome of the JIHS Act! | We appreciate the reviewer’s comment. The sentence is retained as it correctly identifies future evaluation as an essential step given the outcomes described. |
| 471-477  P30-C1 | Consider merging with previous paragraph to avoid repetition | We appreciate the reviewer’s comment. The paragraph beginning "While this study is grounded in Japan's specific legal and administrative context..." has been merged with the preceding paragraph to eliminate repetition while preserving the substantive content. |
| 481-483  P30-C2 | Unclear. Suggest rephrasing. | We appreciate the reviewer’s comment. The sentence has been rewritten for clarity: the revised text specifies that the perspectives captured reflect how policymakers and institutional designers understood the reform in terms of intended function and governance, rather than how it has been experienced by those responsible for operational implementation. |
| 485  P30-C3 | Typo | Accepted. The typo "Nevethless" has been corrected to "Nevertheless." |
| 493-495  P31-C1 | Is it really a "structural gap"? Conceptually, this is not clear. | We appreciate the reviewer’s comment. "Structural gap" has been revised to "gap" to improve conceptual clarity. |
| 507-510  P31-C2 | Noting that this already exists as a concept. It is what the JEE Tool call for at level 5 for Indicator P1 under the Legal Instruments technical area--legal assessments and exercises to test the adequacy of legal frameworks on a regular basis. The GHSA LP AP legal preparedness core elements support this approach. | We appreciate the reviewer’s comment. A new sentence has been inserted referencing the WHO JEE Tool 3.0 Level 5 Indicator P1, which already calls for legal assessments and simulation exercises to test the adequacy of legal frameworks on a regular basis. |
| 513-514  P32-C1 | Why was this? Were the bureaucrats involved not the implementers? Were they not at the technical level? | We appreciate the reviewer’s comment. We agree and have revised this passage. In response to the reviewer's question about why gaps remained between legislative design and practical implementation, we have added clarification that bureaucratic involvement, while present, was primarily at the administrative and coordinative level rather than at the technical and operational level where day-to-day implementation decisions are made. The revised text explains that this limited depth of engagement meant that the perspectives and expertise of technical-level implementers were not sufficiently reflected in the subordinate instruments—such as operational protocols and inter-agency agreements—necessary to translate legal mandates into operational practice. |
| 536-539  P33-C1 | This is why it is important to involve all these key stakeholders/the implementers in the development of the legal framework from the very start. This approach builds buy-in and trust, maximizing effective implementation. | We appreciate the reviewer’s comment. The importance of involving key stakeholders and implementers from the outset of legal framework development is now explicitly stated, noting that this approach builds buy-in and trust to maximize effective implementation. |
| 544-545  P33-C2 | Caution. Not necessarily a new idea. See GHSA LP AP definition of LP and its core elements, namely legal assessment, and also the WHO JEE 3.0's "legal instruments" technical area P1 indicator, level 5. | We appreciate the reviewer’s comment. The revised text references the GHSA LP AP's "legal surveillance" component and the WHO JEE 3.0 to acknowledge that the concept of iterative evaluation is not new, and frames the recommendation accordingly. |
| 548-550  P33-C3 | This concept needs further clarification. Unclear what is meant. | We appreciate the reviewer’s comment. The concept of "learning health systems" has been clarified with the definition by Sheikh and Abimbola, and the text has been revised for greater clarity. |
| 552  P33-C4 | Formatting issue. Seems like a (sub) heading. | Accepted. The formatting of ".Expected Outcomes and International Relevance" has been corrected. It is now properly formatted as a sub-heading. |
| 553  P34-C1 | this is contained in "legal assessment" (one of LP's core elements) per the GHSA LP AP | We appreciate the reviewer’s comment. "Evaluation model" has been revised to "legal assessment process, consistent with the legal assessment  component of legal preparedness as defined by the GHSA LP AP." |
| 561  P34-C2 | The point of this section and how it is addressed is not clear to me. Doesn't seem to sufficiently present alternative interpretations. Also, "alternative interpreations" of what? Evaluation model? Analysis of the JIHS Act? Alternative arguments? | We appreciate the reviewer’s comment. The entire "Alternative Interpretations" section has been removed as it did not sufficiently present alternative interpretations and was largely redundant with content addressed elsewhere in the manuscript. |
| 562-565  P34-C3 | These are implementation issues - tied or not to the adequacy of the JIHS Act | We appreciate the reviewer’s comment. The "Alternative Interpretations" section, including the list of implementation concerns, has been removed. The substantive points have been integrated into the main discussion where appropriate. |
| 578-580  P35-C1 | Flagging this again. See previous comment about concerns about the messaging. A reframing is strongly recommended, which can make the argument stronger. | We agree and have revised this sentence. The original framing, which suggested that legal frameworks alone are insufficient, has been reframed to avoid undermining the role of legal frameworks in crisis response. The revised text retains the "however" construction but redirects the argument: rather than implying that legal frameworks are inadequate, it now emphasizes that operational adequacy depends on the quality of the design process—particularly the meaningful consultation of technical-level implementers from the outset—and on the establishment of ongoing mechanisms to test, validate, and adapt frameworks through practice. Additional context has also been added to note that policy challenges were comprehensively mapped through expert advisory councils prior to enactment, and that the JIHS Act, consistent with Japanese framework legislation, delineates broad institutional parameters subject to continuous refinement through subordinate regulations. This reframing strengthens the argument by grounding the implementation challenge within legal preparedness scholarship rather than positioning it as a limitation of legal frameworks per se. |
| 581-586  P35-C2 | Noting again that is not a new concept. It is embedded already in the WHO JEE 3.0 and the GHSA LP AP definition. | We appreciate the reviewer’s comment. The revised text acknowledges alignment with the legal assessment and legal surveillance elements of the GHSA LP AP framework and consistency with WHO JEE Tool 3.0 standards. |

**Response to the Editorial Office (Technical Checks)**

**Data Availability**

In response to the editorial office's data availability requirement, we clarify that the data underlying this study consist of key informant interview transcripts containing potentially identifying and sensitive information, which cannot be made publicly available for ethical reasons. These restrictions were imposed by the Ethics Review Committee (Institutional Review Board) of the Japan Institute for Health Security (JIHS; Approval No. JIHS-S-005089-00), which approved the study. A de-identified minimal data set is available to researchers who meet the criteria for access to confidential data, upon request to the following non-author institutional contact: Secretariat of the Ethics Review Committee, JIHS (Email: kenkyu-shinsa@jihs.go.jp; Tel: +81-3-3202-7181; Address: 1-21-1 Toyama, Shinjuku-ku, Tokyo 162-8655, Japan). We have updated the Data Availability statement in the Editorial Manager system accordingly.

**Figure Copyright**

In response to the editorial office's figure copyright requirement, we confirm that all figures in the manuscript were created by the authors and do not reproduce any previously published or third-party copyrighted material. Figure 1 is an original conceptual figure prepared by the authors. Figure 2 was newly created by the authors using original icons and graphical elements produced for this study; it illustrates the institutional relationships based on publicly available information from the Cabinet Agency for Infectious Disease Crisis Management (CAICM) website and does not reproduce any copyrighted image. Accordingly, no third-party permission is required, and all figures are fully compatible with publication under the CC BY 4.0 license.
