# Supplementary material for: Legal design for establishing a novel parastatal public health institute – Japan Institute for Health Security: A case study
Source: PLOS Glob Public Health. 2026 Aug 26;6(8):e0007088. doi: 10.1371/journal.pgph.0007088 (PMC13514060; doi:10.1371/journal.pgph.0007088)
Supplement: Peer review history

# Peer review: Decision Letter 0

**DOI:** 10.1371/journal.pgph.0007088.r001

**Authors:** Benjamin Meier

23 Apr 2026

PGPH-D-26-00460

Legal design for establishing a novel parastatal public health institute – Japan Institute for Health Security: A case study

PLOS Global Public Health

Dear Dr. Saito,

Thank you for submitting your manuscript to PLOS Global Public Health. After careful consideration, we feel that it has merit but does not fully meet PLOS Global Public Health’s publication criteria as it currently stands. Therefore, we invite you to submit a revised version of the manuscript that addresses the points raised during the review process.

Please submit your revised manuscript by Jun 07 2026 11:59PM. If you will need more time than this to complete your revisions, please reply to this message or contact the journal office at globalpubhealth@plos.org. When you're ready to submit your revision, log on to https://www.editorialmanager.com/pgph/ and select the 'Submissions Needing Revision' folder to locate your manuscript file.

Please include the following items when submitting your revised manuscript:

- A letter that responds to each point raised by the editor and reviewer(s). You should upload this letter as a separate file labeled 'Response to Reviewers'.
- A marked-up copy of your manuscript that highlights changes made to the original version. You should upload this as a separate file labeled 'Revised Manuscript with Track Changes'.
- An unmarked version of your revised paper without tracked changes. You should upload this as a separate file labeled 'Manuscript'.

Guidelines for resubmitting your figure files are available below the reviewer comments at the end of this letter.

As the corresponding author, your ORCID iD is verified in the submission system and will appear in the published article. PLOS supports the use of ORCID, and we encourage all coauthors to register for an ORCID iD and use it as well. Please encourage your coauthors to verify their ORCID iD within the submission system before final acceptance, as unverified ORCID iDs will not appear in the published article. *Only*  the individual author can complete the verification step; PLOS staff *cannot*  verify ORCID iDs on behalf of authors.

We look forward to receiving your revised manuscript.

Kind regards,

Benjamin Mason Meier, J.D., LL.M., Ph.D

Academic Editor

PLOS Global Public Health

Journal Requirements:

1. Please send a completed 'Competing Interests' statement, including any COIs declared by your co-authors. If you have no competing interests to declare, please state "The authors have declared that no competing interests exist".

1. Please amend your detailed Financial Disclosure statement. This is published with the article. It must therefore be completed in full sentences and contain the exact wording you wish to be published.
  If you did not receive any funding for this study, please simply state: “The authors received no specific funding for this work.”

1. Please provide separate figure files in .tif or .eps format.
  For more information about figure files please see our guidelines:
https://journals.plos.org/globalpublichealth/s/figures
https://journals.plos.org/globalpublichealth/s/figures#loc-file-requirements

1. Please insert an Ethics Statement at the beginning of your Methods section, under a subheading 'Ethics Statement'.
  1) The name(s) of the Institutional Review Board(s) or Ethics Committee(s)
  2) The approval number(s), or a statement that approval was granted by the named board(s)
  [3) (for human participants/donors) - A statement that formal consent was obtained (must state whether verbal/written) OR the reason consent was not obtained (e.g. anonymity). NOTE: If child participants, the statement must declare that formal consent was obtained from the parent/guardian.]

1. We note that you have indicated that there are restrictions to data sharing for this study. For studies involving human research participant data or other sensitive data, we encourage authors to share de-identified or anonymized data. However, when data cannot be publicly shared for ethical reasons, we allow authors to make their data sets available upon request. For information on unacceptable data access restrictions, please see http://journals.plos.org/plosone/s/data-availability#loc-unacceptable-data-access-restrictions.
  Before we proceed with your manuscript, please address the following prompts:
  a) If there are ethical or legal restrictions on sharing a de-identified data set, please explain them in detail (e.g., data contain potentially identifying or sensitive patient information, data are owned by a third-party organization, etc.) and who has imposed them (e.g., a Research Ethics Committee or Institutional Review Board, etc.). Please also provide contact information for a data access committee, ethics committee, or other institutional body to which data requests may be sent.
  b) If there are no restrictions, please upload the minimal anonymized data set necessary to replicate your study findings to a stable, public repository and provide us with the relevant URLs, DOIs, or accession numbers. Please see http://www.bmj.com/content/340/bmj.c181.long for guidelines on how to de-identify and prepare clinical data for publication. For a list of recommended repositories, please see https://journals.plos.org/plosone/s/recommended-repositories. You also have the option of uploading the data as Supporting Information files, but we would recommend depositing data directly to a data repository if possible.
  Please update your Data Availability statement in the submission form accordingly.

If the reviewer comments include a recommendation to cite specific previously published works, please review and evaluate these publications to determine whether they are relevant and should be cited. There is no requirement to cite these works unless the editor has indicated otherwise.

Additional Editor Comments (if provided):

[Note: HTML markup is below. Please do not edit.]

Reviewers' comments:

Reviewer's Responses to Questions

**Comments to the Author**

1. Does this manuscript meet PLOS Global Public Health’s publication criteria?

Reviewer #1: Partly

Reviewer #2: Yes

**********

2. Has the statistical analysis been performed appropriately and rigorously?

Reviewer #1: Yes

Reviewer #2: N/A

**********

3. Have the authors made all data underlying the findings in their manuscript fully available (please refer to the Data Availability Statement at the start of the manuscript PDF file)?

The PLOS Data policy

Reviewer #1: Yes

Reviewer #2: Yes

**********

4. Is the manuscript presented in an intelligible fashion and written in standard English?

Reviewer #1: Yes

Reviewer #2: Yes

**********

Reviewer #1: I enjoyed reviewing this manuscript. Overall, the research is certainly original, and provided that critical technical revisions are incorporated to ensure technical soundness, its publication (including its promotion) would be of great value to legal preparedness work in both Japan and countries around the world–the space would benefit greatly from the research and findings, including the interviews. The very tracking of the various steps that it took to establish the JIH is itself a contribution to the world, particularly for countries who are looking to do the same. There is currently a need to further promote the importance of legal preparedness as a critical capacity for effective public health emergency preparedness and response. In general, the piece promises to be a value add. However, critical revisions are needed, including a revisit of the proposed “evaluation model.” As further elaborated below, the manuscript presents it as a new approach, but the practice has already been conceptualized by others, including the WHO Joint External Evaluation Tool 3.0 (Indicator P1, Level 5) and the Global Health Security Agenda (GHSA) Legal Preparedness Action Package’s (GHSA LP AP) definition of legal preparedness. That being said, with some reframing and adjustments to the argument (such as by promoting this approach without claiming that it as a new approach), the manuscript may not need substantial reworking in that section. The manuscript could also benefit from additional literature on legal preparedness in the context of health emergencies, particularly post COVID-19 pandemic.

In reviewing this piece, it is clear that there is also a misconception of what legal preparedness entails and how legal frameworks should be designed to ensure effective implementation for high impact on population health. While the research and analysis does demonstrate this need for legal assessments (particularly ongoing), there are critical revisions that need to be made as well as framings that would help strengthen it. I have provided comments throughout the manuscript, which is attached. If allowed, I would be more than happy to connect with the authors to help inform their revisions to more accurately capture what has become a more widely accepted understanding of legal preparedness, its components, and objectives.

Starting with the critical recommended revisions, it appears that the desk research has not been as comprehensive. The analysis requires establishing an understanding of legal preparedness from the start, and one based on existing literature. As written, there is no definition provided that helps frame the various arguments made throughout as well as its main argument. For this, I highly suggest directly referring to and incorporating the GHSA LP AP’s definition of “legal preparedness” in the context of public health emergencies, developed in 2021 to provide a common understanding of this capacity following the COVID-19 pandemic. Governments, academics, and civil society organizations participated in the development of this definition, which has been used by experts globally. It can be found here: https://globalhealthsecurityagenda.org/wp-content/uploads/2024/08/Final_May2023_Legal-Preparedness-Definition.pdf. In addition to the definition itself, because the piece discusses what is being proposed as an evaluation model, I specifically refer the authors to the five core elements of legal preparedness (also found in the same document: legal analysis (consisting of legal mapping, legal assessment, and legal surveillance), legal frameworks, capacity, coordination, and implementation tools. With this, I’d like to caution the authors that the concept of a reiterative process and using simulation exercises to evaluate legal frameworks over time has already been proposed by the LP AP and others, including WHO. The latest version of the WHO Joint External Evaluation (JEE) tool (3.0) (https://iris.who.int/server/api/core/bitstreams/f9a1d72f-d8e4-4abe-900f-040829de686d/content) incorporates this idea in the “legal instruments” technical area’s indicator P1. Without acknowledgement of these legal preparedness advancements and also the presentation of an evaluation framework as a newly proposed ideal (already understood as legal surveillance) risks undermining the credibility of the authors’ thoroughness in their research and analysis. I’m not concerned that the authors would not be able to adapt their analysis in light of this–if anything, I believe that analysis building off of these precedents would help promote these concepts further and make their piece even stronger. Also recommend referring to “"Legal Preparedness as a Foundation for Global Health Security" (2025) (https://pubmed.ncbi.nlm.nih.gov/40485256/) and ”Strengthening legal preparedness and response within the global health emergency framework: the role of the GHSA Legal Preparedness Action Package” (2024) (https://www.elgaronline.com/view/journals/jghl/1/1/article-p88.xml) among other more recent literature post COVID.

The authors state that “Future evaluation of JIHS performance will determine whether legal frameworks deliver intended improvements in pandemic preparedness and response” (lines 48-49). This conclusion raises serious concerns that require reconsideration. It risks undermining the value of legal frameworks in pandemic preparedness and response, particularly for the less versed. What is more, this assumes that the JIHS Act was well designed, but based on what has been described in the manuscript, there are major gaps resulting from an incomplete consultation with key actors, particularly the implementers, which keeps it from being effective. For a legal framework to "determine" whether legal frameworks deliver intended improvements in pandemic preparedness and response, the legal framework would have to be well designed, but that doesn’t seem to be case. Also, it is dangerous to claim that the JIHS Act to represents all legal frameworks and therefore should be the litmus test for whether legal frameworks are effective in pandemic preparedness and response.

Additionally, the authors argue that there is “tension between structural authority and motivational legitimacy,” which is guided by interviewees’ observations about the JIHS Act implementation, namely: “legal frameworks are insufficient for operationalization, requiring inter-agency trust and cultural integration between merged institutions” and “law alone cannot move people; motivation and a shared mission are essential” (lines 284-285). The authors frame the latter as a “deeper truth.” The authors also point out that “[s]ome informants expressed concern that the legal merger might not automatically translate into cultural and operational integration.” Based on how legal preparedness has been understood and defined by experts, I would observe that the experience of Japan clearly shows the unintended and unfortunate consequences that results from designing a legal framework that is not informed by the mission, motivation, and mission from the start, which significantly comes from the input from technical-level experts who are most familiar the culture at the implementation level (this is critical to ensure that a legal framework response to reality). The authors’ interviewees seem to be limited to higher level government officials and do not include technical-level individuals who are most familiar with the day-to-day implementation. It is also not apparent that the JIHS Act development involved meaningful consultations with technical-level officials or communities to better understand the reality closer to the ground. In other words, when designing legal frameworks, the shared mission, good practices, and lessons learned (as underwood by the implementers) should be front and center and from the very start. Otherwise, the implementation of the legal framework results in the JIHS experience as described by the authors. This is not something that the authors have considered, and it is essential to posit this possibility. As written, the analysis risks undermining the importance of legal preparedness based on misconceptions of how legal frameworks should be designed. I strongly recommend that the authors reframe how these observations and conclusions are framed. Further, per the legal preparedness core elements (as understood by the LP AP), the remedy would be the “legal assessment” and “legal surveillance” (including exercises to test) of the JIHS legal framework in the coming years toward improving it over time.

At the same time, building off of the last point, I found this manuscript a fascinating case study of how health security, anthropology, and the law intersect and the types of outcomes that certain approaches can yield. Essentially, if legal frameworks are not informed by the context, culture, and key stakeholders (e.g., implementers, those directly affected by the legal framework (communities)), its effectiveness can be questionable. It may not be possible to elaborate this perspective, but I did want to make sure I pointed this out. I have designed and taught a course on this subject, and would be happy to connect with the authors should they be interested in a greater understanding of this point.

Ultimately, What this experience and conclusions point to is a lack of legal preparedness expertise in Japan. There seems to be insufficient knowledge among government officials of the field. There could be important opportunities to strengthen capacity in this area for legal frameworks to meet reality and the needs.

Generally, the article is written in standard English. However, in some sections, particularly ones that are trying to introduce abstract concepts, some statements are hard to follow (e.g., lines 421-424). I strongly recommend revising with plain English to the extent possible. Otherwise, it's easy to get lost on the takeaway that the article is trying to convey here.

Reviewer #2: The article provides a methodologically and ethically rigorous analysis of the legal and institutional reform processes that led to the establishment of the Japan Institute for Health Security (JIHS) as part of Japan’s effort to overcome the pitfalls and challenges identified in its national health security system in response to the COVID-19 pandemic. Through documental review of legislation, policies and grey literature, complemented by qualitative analysis of data from key informant interviews (KII), the paper reviews and analyses the JIHS legal status, governance, structure, authorities, roles and functions, and assesses how these legal and institutional reforms contributed to the transformation of the national health security system in Japan. Based on this analysis, the article claims that the JIHS legal reform process has strengthened Japan’s infectious disease governance. At the same time, however, the paper also acknowledges the limitations of legal frameworks alone in ensuring full country readiness to prevent and respond to public health crises, and therefore expands the analysis from the legal assessment to real-word observation. In addition to the legal analysis, hence, the paper touches upon complex and sensitive issues about the role of the law in strengthening countries’ public health security; including the tension between structural authority and motivational legitimacy; the challenges of transforming legislative authorities and powers into effective operationalization; the complex relationship between science and policy; the need for capacity building and continued institutional learning; the important human and cultural dimension underpinning legal reforms. Ultimately, the paper suggests an evaluation model to validate the ‘real-world effectiveness’ of these legal reforms and assess whether and how the reformed legal frameworks concretely deliver on the promise of strengthening Japan’s preparedness to future public health crises.

The paper contributes to enhancing the understanding of national public health governance frameworks for public health emergency preparedness and response in Japan. In addition to the context-specific value of this case study, importantly, the article also contributes to the refinement and operationalization of the concept of legal preparedness through its application and verification against context-specific needs and practice, offering transferable and generalizable considerations and recommendations that may provide relevant lessons for other countries and in different legal settings.

The paper structure is solid and sound, and the manuscript is well organized and reader-friendly. Literature review provides a limited account of the existing grey literature published on this topic, but a solid review of primary sources. Tables and figures help the reader summarize key information from the legal analysis. Data collection method is adequate and appropriate, and review of data was purposively performed through a functional approach to respond to the specific research questions and overall provide evidence-based conclusions to the assessment of JIHS legal readiness through an analysis of legal authorities and powers. The methodology for the KII, based on the Braun and Clarke's thematic analysis framework, is sound and ethically rigorous, in compliance with ethical standards. It provides added value to the analysis of legal documents to better understand strengths and pitfalls of the legal structure in the practical operationalization of the JIHS. Restriction on public access to data for KIIs is justified based on ethical limitations as explained by authors. The legal analysis is well performed, it clearly identifies roles and functions of the two pre-existing institutions (NIID and NCGM), and accurately describes how these roles and functions were then subsumed in the JIHS conceptual framework and subsequent regulation, and relevant authorities and powers are appropriately clear and well-defined. Overall, it provides a good exercise of legal mapping for public health emergency governance. The discussion of the findings flows logically from the data collected, and leads nicely to the policy recommendations provided. Conclusions are clear and sound.

Suggestions for future improvement:

Although overall the paper is technically rigorous and meets the scientific and ethical standards for publication in line with PLOS criteria as such, the Authors may wish to consider the following suggestions to further expand the scope of the analysis:

- Expand the analysis of the institutional and legal ecosystem of public health governance, including considerations on the relationship between JIHS beyond public health institutions and authorities, including in the areas of food, environmental, climate, animal health governance, among others.

- Address the alignment of the national reform process towards the establishment and operationalization of JIHS against global health governance processes and international standards (such as International Health Regulations)

- Further elaborate on the need for organizational learning and capacity building for institutional strengthening and cross-agency collaboration

**********

what does this mean?). If published, this will include your full peer review and any attached files.

**Do you want your identity to be public for this peer review?**  If you choose “no”, your identity will remain anonymous but your review may still be made public.

For information about this choice, including consent withdrawal, please see our Privacy Policy

Reviewer #1: **Yes:** Ana S. Ayala

Reviewer #2: No

**********

[NOTE: If reviewer comments were submitted as an attachment file, they will be attached to this email and accessible via the submission site. Please log into your account, locate the manuscript record, and check for the action link "View Attachments". If this link does not appear, there are no attachment files.]

Attachment Submitted filename: PGPH-D-26-00460-ASA.pdf

# Author comment: Author response to Decision Letter 1

**DOI:** 10.1371/journal.pgph.0007088.r002

19 Jul 2026

Attachment Submitted filename: ANNEX Response to comments.docx

# Editor report: Decision Letter 1

**DOI:** 10.1371/journal.pgph.0007088.r003

**Authors:** Benjamin Meier

26 Jul 2026

Legal design for establishing a novel parastatal public health institute – Japan Institute for Health Security: A case study

PGPH-D-26-00460R1

Dear Dr. Saito,

We are pleased to inform you that your manuscript 'Legal design for establishing a novel parastatal public health institute – Japan Institute for Health Security: A case study' has been provisionally accepted for publication in PLOS Global Public Health.  This scholarship will prove crucial to the rapidly evolving academic field of Global Health Law and support legal preparedness under the Global Health Security Agenda.

Before your manuscript can be formally accepted you will need to complete some formatting changes, which you will receive in a follow up email. A member of our team will be in touch with a set of requests.

Please note that your manuscript will not be scheduled for publication until you have made the required changes, so a swift response is appreciated.

IMPORTANT: The editorial review process is now complete. PLOS will only permit corrections to spelling, formatting or significant scientific errors from this point onwards. Requests for major changes, or any which affect the scientific understanding of your work, will cause delays to the publication date of your manuscript.

If your institution or institutions have a press office, please notify them about your upcoming paper to help maximize its impact. If they'll be preparing press materials, please inform our press team as soon as possible -- no later than 48 hours after receiving the formal acceptance. Your manuscript will remain under strict press embargo until 2 pm Eastern Time on the date of publication. For more information, please contact globalpubhealth@plos.org.

Thank you again for supporting Open Access publishing; we are looking forward to publishing your work in PLOS Global Public Health.

Best regards,

Benjamin Mason Meier, J.D., LL.M., Ph.D

Academic Editor

PLOS Global Public Health

***********************************************************

Reviewer Comments (if any, and for reference):
